# Magnitude and outcome of road traffic accidents among patients admitted in dessie town governmental hospitals, Northeast Amhara, Ethiopia, 2022

**DOI:** 10.1186/s12873-024-01047-1

**Published:** 2024-07-29

**Authors:** Fatuma Seid Degu, Adem Hussein Endris, Samuel Anteneh Ayele, Natnaiel Grima Melkie, Mitaw Girma Kenbaw, Mekuriaw Wuhib Shumye, Missale Kassahun Hirpo, Atrsaw Dessie Liyew, Mandefro Assefaw Geremew, Prem Kumar

**Affiliations:** 1 Department of Nursing, College of Medicine and Health Sciences, Wollo University, PO Box 1145, Wollo, Ethiopia; 2 Department of Midwifery, College of Medicine and Health Sciences, Wollo University, Wollo, Ethiopia

**Keywords:** Dessie, Hospitals, Magnitude, Road traffic accidents, Outcome

## Abstract

**Background:**

Road traffic accidents(RTA) are a major public health problem worldwide, accounting for almost 1.24 million deaths per year and it is the number one cause of death among those aged group 15–29 years. Even though there are great benefits from access to road transportation there also poses a great challenge in the individual’s daily activities ranging from minor injury to death.

**Objective:**

This study aimed to assess the magnitude and outcome of road traffic accidents among patients admitted in Dessie Town Governmental Hospitals, Northeast Amhara, Ethiopia, 2022.

**Methods:**

A five-year hospital-based retrospective descriptive cross-sectional study design was conducted among 377 road traffic accident patients admitted to Dessie Town Governmental hospitals. Data were collected by simple random methods based on patient chart reviews from June 7/, 2022 to May 23/ 2017 using a checklist adapted from the WHO standard hospital-based road traffic accident questionnaires after obtaining consent from the concerned authority. EPI-Data software version 7.2 for data entry and SPSS version 25 for statistical analysis were used. Descriptive and inferential statistics were used. Statistical significance was declared at a p-value of < 0.05 with an adjusted odds ratio (AOR) and a 95% confidence interval (CI) in the final multinomial logistic regression model.

**Results:**

The magnitude of road traffic accidents was 59%, using of logistic multi nominal logistic regression we found results such that, road traffic victims who had unstable vital signs at admission (AOR = 6.4,95% CI; 2.5–16.6), didn’t get prehospital treatment (AOR = 9.3,95% CI; 4–20), and severe injury (AOR = 9, 95% CI;7-15.4), had a Glasgow coma scale of 3–5 (AOR = 5.2,95% CI; 1.4–20) were found predictors for death were as unstable vital signs at admission (AOR = 3.79,95%CI;2.1–6.8), Doesn’t get prehospital treatment (AOR = 2.8, 95% CI; 1.4–5.7), Hospital stay for one to two months duration (AOR = 6,95% CI;2.3–15), and greater than two months duration (AOR = 6.5,95%CI;2.5–17) were found predictors for disability among road traffic victims.

**Conclusions and recommendations:**

Road traffic accidents constitute a major public health problem in our setting and contribute significantly to excessively high morbidity and mortality. Unstable vital signs at admission, Client doesn’t get prehospital treatment, severely injured client, and had a Glasgow coma scale of 3–5 were found predictors for death were as an unstable vital sign at admission, Client doesn’t get pre-hospital treatment, Hospital stays for one to two months duration, and greater than two months duration were found predictors for disability among road traffic victims.

**Supplementary Information:**

The online version contains supplementary material available at 10.1186/s12873-024-01047-1.

## Introduction

### Background

Road transportation has a direct connection with the day-to-day activities of people, especially in large cities where the distance to be traveled is too far to cover on foot or by bicycle within a reasonable time. The Global Status Report on Road Safety 2023 shows that the number of annual road traffic deaths has fallen slightly to 1.19 million [1].

According to the report on road traffic injury showed that the number of road traffic injuries has continued to rise in the whole world, but there has been an overall downward trend in road traffic deaths in high-income countries since the 1970s and an increase in many of the low-income and middle - income countries. Deaths related to road traffic injury (RTI) are predicted to increase by 83% in low-income and middle-income countries and to decrease by 27% in high-income countries [2]. The severity of road traffic accidents is also likely to be much greater in Africa than anywhere else because many vulnerable road users are involved, poor transport conditions such as lack of seat belts, overcrowding, and hazardous vehicle environments. The poor reporting system has also masked the magnitude of the problem in the Africa region. The lack of pre-hospital and hospital emergency care after accidents makes the outcome of car accidents the worst in Africa. African countries had the highest mortality rate, with 28.3 deaths per 100,000 populations, and, In Ethiopia a road traffic fatality rate of 37 per 100,000 population [3, 29]. Ethiopian Federal Police Commission recorded 15,034 road accidents in 2021, resulting in 4,161 deaths, surpassing the World Health Organization’s 2013 record of 4,984.3 deaths per 100,000 vehicles per year[4]. Over the 45 months from September 2013 to May 2017, 3385 road traffic accidents were reported in Amhara Region. The average monthly number of accidents was 76, with the highest being 108 and the lowest being 43 [28]. Dessie Town took the third rank, following Gondar and Bahir Dar city 86.3%,54.8%, and 48.5% respectively [5].

**Fig. 1:**
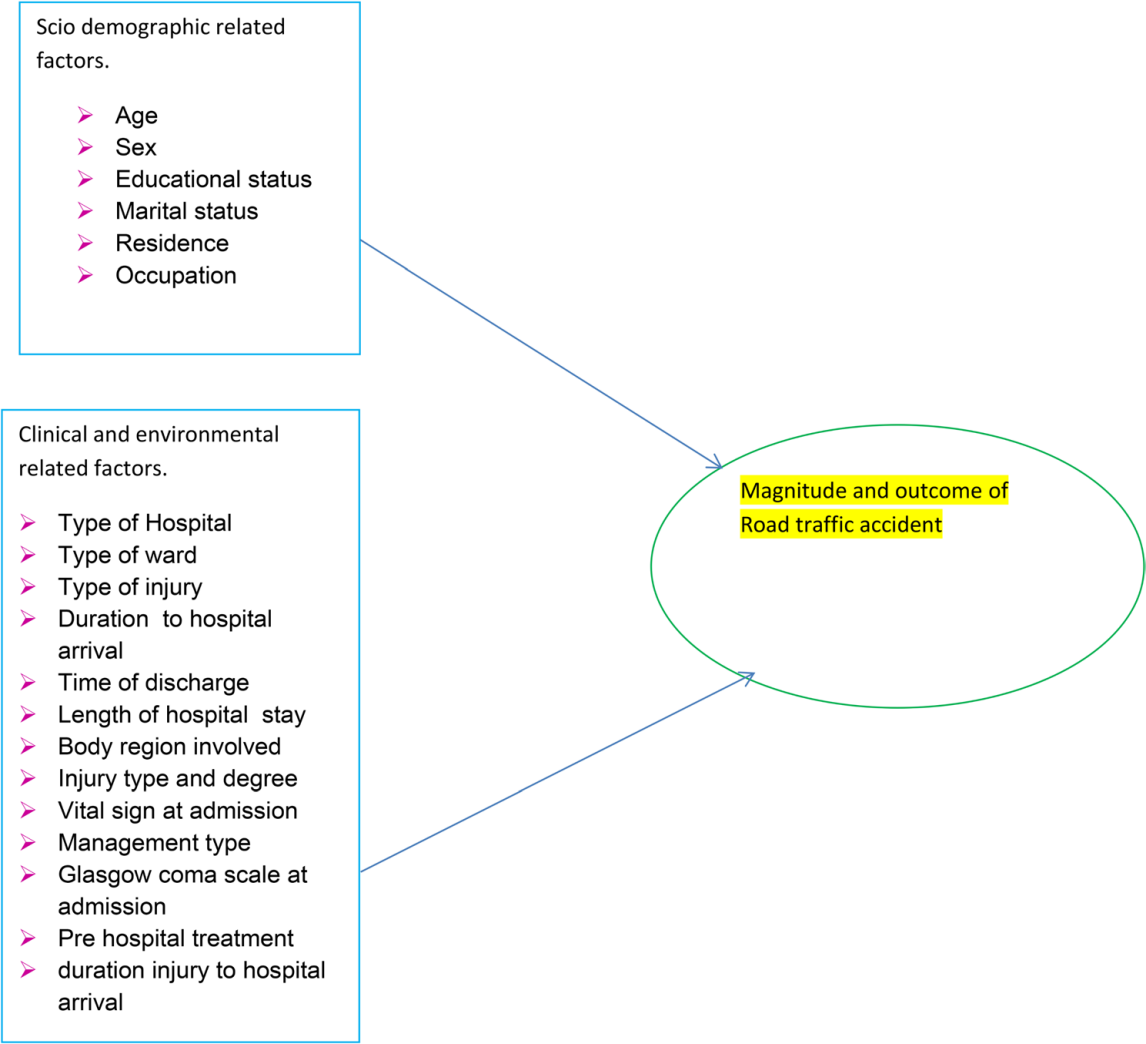
Conceptual framework shows the relationship between dependent and independent variables

Scholars suggest factors contributing to fatality rates in road traffic victims include age (> 60 years), systolic blood pressure, Glasgow coma scale, head injury, time to reach a health facility, patient condition, hospital days, abdominal injury, transfer status, blood transfusion, ICU admission [6–9]. Delay to come to the hospital (over 24 h), the severity of injuries, and management types [10] were significant indicators of death among the road traffic victims. According to many studies notify that, Road traffic accidents affect not only the health of individuals but also their family members, as it can drive households into poverty when they struggle to cope with the long-term consequences of the events, such as the costs of medical care, rehabilitation and loss of family’s breadwinners. RTAs have substantial adverse effects on national health systems as well, many of which already have suffered from woefully inadequate levels of resources [11]. Rescue the trapped casualties, looking for breathing, heart function, and consciousness, controlling bleeding and fractures, and moving the casualty to the closest hospital were considerable treatment measures [12] (Figure 1).

Currently, there is limited literature to generalize the country’s context. However, no study has addressed the trends of road traffic accidents for at least five years of duration. Adding to this, similarly, previous researchers used logistic regression only, whereas the current study used multinominal logistic regression and included the new variable disability and outcome variables, and this study insight into hospital administrative units’ national road safety commissions, motor traffic, and transport units, and other stakeholders to develop effective treatment responses and strategies for road accident admission victims. It also provides evidence on effective road traffic accident prevention, patient care, and rehabilitation, serving as a baseline for future research. Hence the study aimed to assess the magnitude outcome of road traffic accidents among patients admitted to Dessie town governmental hospitals.

## Method and materials

### Study design, area, and period

The hospital-based retrospective descriptive cross-sectional study design was conducted at Dessie Town governmental hospitals; Dessie Town is one of the eleven zones in Amhara Regional state and the city of the South Wollo Zone. It is located at a distance of 401 km from Addis Ababa. According to the 2007 Central Statistical Agency report, Dessie has 285,530 populations in 2021/2022, of which 49.5% are men. In 2019/2020, there were 8 health centers, 8 health posts, 2 government hospitals, 3 private hospitals, 38 private clinics, 55 private drug stores, and 4 private diagnostic laboratories. Dessie Town governmental hospitals as the three main wards in the hospital, the orthopedic, medical, and surgical wards, together treat about 3600 patients annually. There are currently 39 beds in the surgical ward with five general surgeons and 19 staff members and 36 beds in the orthopedic ward with two orthopedics and 12 staff members providing care [13]. Boru Media General Hospital is located 20 km from Dessie Town and has different departments and wards surgical wards have 10 beds and the orthopedic has 7 beds with 3 surgeons and 12 staff was currently giving services. Moreover 409 road traffic patients were visited per month in the respective study area. The study was conducted from June 7/2022-23/ 2022.

### Source populations

All patients who experience RTA traumatic injury are admitted to Dessie Town governmental hospitals.

### Study population

All patients admitted with road traffic accidents to Dessie Town governmental Hospitals during study periods.

### Sample size and sampling method

Sample size determination using a single population formula

$$\:{\left(za/2\right)}^{2}\frac{p\left(1-p\right)}{d2}$$

P = proportion = 33.6% (14).

$$\:\left(\mathbf{Z}\frac{\varvec{\alpha\:}}{2}\right)2$$=1.96

D = Degree of precession = 5%.

**Fig. 2:**
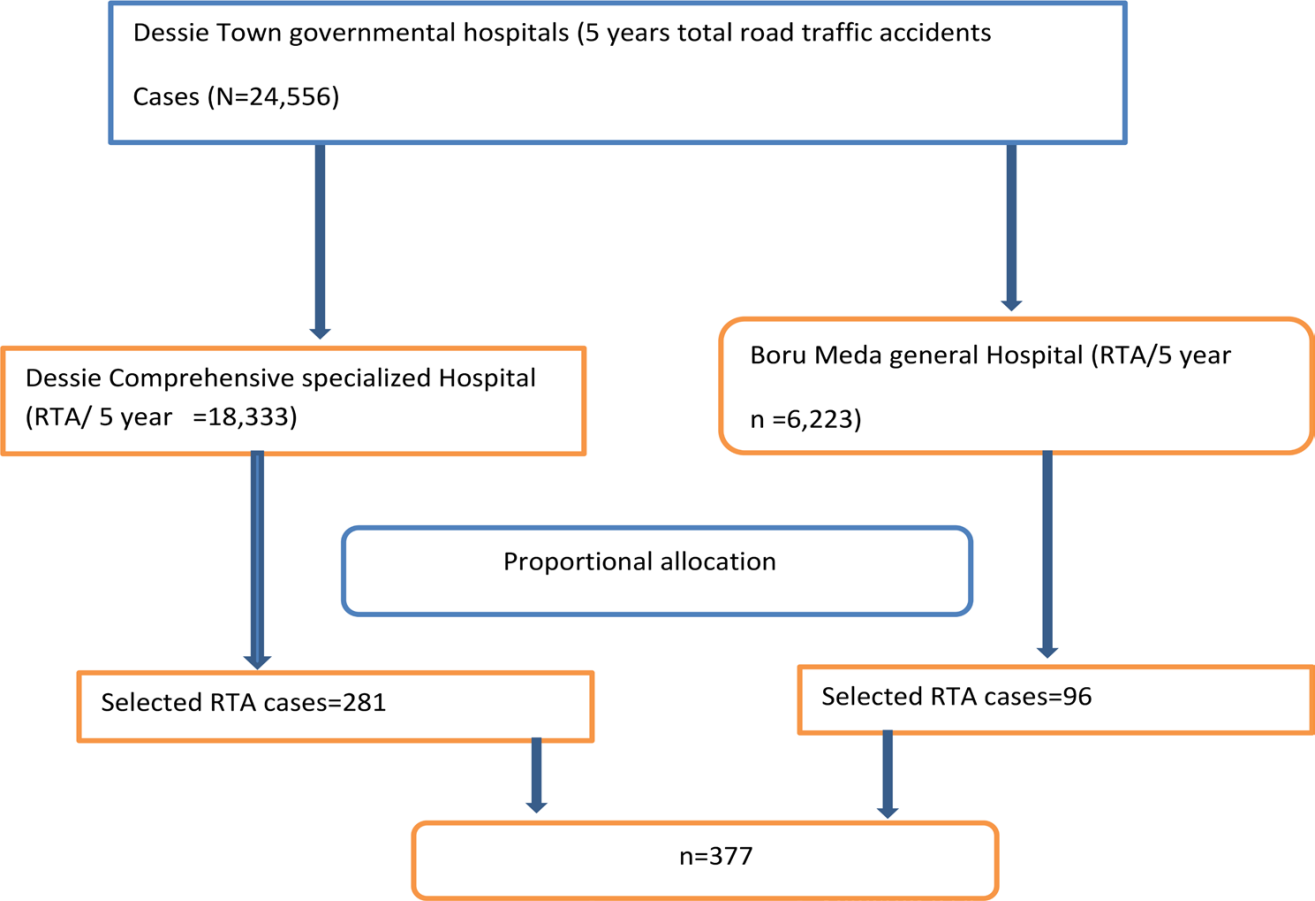
Sampling procedure about the of road traffic accident, and treatment outcome at Dessie Town governmental Hospitals, North east Ethiopia, 2022

By adding a 10% non-response rate the final sample size was 377 (Figure 2).

Sampling technique: A simple random sampling technique was used to select study participants.

### Variables of the study

Dependent variables: Magnitude of Road Traffic Accidents.

Independent variables.

### Socio-demographic characteristics

Age, Sex, Educational status, marital status, Residence, and occupation.

### Environmental and clinical related factors

The factors such as Type of injury and Hospital, Length of hospital stay, body area/part involved degree of injury as mild, moderate and severe, vital signs at admission, management type, Glasgow coma scale at admission, and comorbidity disease.

### Data collection tools and procedures

Data was collected using a checklist adopted from the WHO hospital-based standard with 15 main questionnaires of road traffic accidents by 3 BSc nurses and one supervisor after they were trained by the principal investigator for one working day before the actual data collection date. To get the patient’s primary files from the card room, the card number was first taken from the log books in each department, including the emergency room, operating room, surgical, and medical inpatient records. To gather the necessary information for the study participants, the patient’s card’s medical record number (MRN) was fully listed next to it, along with the relevant study periods. Then all MRNs were cross-checked across each department and unit to avoid any duplication. Finally, based on the inclusion criteria of the study cards which had all variables for the study were selected based on simple random techniques after a proportional allocation number of cases per year. Then all variables like pre-hospital care, body part injured, types of diagnosis, treatment modality, treatment outcome, and other variables were collected from chief complaint, history of present illness, progress, admission, and discharge note.

### Data processing and analysis

The data was entered by Epidata Version 7.2 and analyzed using SPSS version 25. Data cleaning was performed to check for frequencies, accuracy, consistency and missed values and variables. Any error identified during data entry was corrected after revision of the original completed checklist.

Statistical significance was declared at a p-value of < 0.05 with an adjusted odds ratio (AOR). To explain the study population about relevant variables descriptive like percentage, and frequency, and analytical statistics like, tables, and binary and multivariate logistic regression were used to present data and to show the relationship between the dependent and independent variables in the study. All explanatory variables enter into the multivariate logistic regression model to control the possible effect of confounders and by using the backward stepwise regression method. Finally, the variables had an independent association with treatment outcome, death, and disability was declared based on 95% CI and p-value < 0.05. Model fitness was checked by using the Hosmer and Lemeshow’s goodness of fit test which was 0.954.

### Data quality control and assurance

A pre-test was done with 5% of the sample size at Kemissie General Hospital, Ethiopia. The data collectors and supervisor were trained for 2 days on how to collect the data from the particular participants. The progress of data collection was scrutinized by the supervisor every other day. Model fitness was confirmed by the Hosmer and Lemeshow test, and it was 0.954.

### Operational definition

#### Injury

Physical damage to the body, intentionally or unintentionally [15].

#### Degree of injury

The degree of the injury is determined by the extent of the injury, including superficial, moderate, and severe injuries, which require skilled treatment [14].

#### RTA

collisions involving two or more automobiles with automobiles as well as automobiles with people, automobiles with animals, and, automobiles with immovable or fixed objects [15].

#### RTA death

Any patient or victim admitted and starting treatment at selected healthcare facilities [29].

#### First aid

It is the implementation of measures of the immediate care taken of the lives of people with traumas and illnesses until they are given professional medical assistance [30].

#### Good treatment outcome

The client remained discharged with improvements and/or deprived of impediments like hearing loss, vision loss, amputations, and so on. [15]

#### Poor treatment outcome

If the patient remained discharged through complications or transferred/shifted to a tertiary or specialized health setup, or deceased in the hospital [15].

#### Death at arrival

It refers to the death of a patient when brought to the hospital and /or within an hour of existence at the hospital [15].

#### Passenger

Individuals who travel in a vehicle or transport system [31].

#### Pedestrian

A person transporting themselves in the most natural expression of what it means to be human [32].

## Results

### Socio-demographic characteristics of the study participants

A total of 377 participants were enrolled in this study, making a 100% response rate.

The mean ages of the study participants were 27.34 years with (SD, 12.86). More than half of the study participants, 237 (62.9%) were male. Regarding their marital status, 129 (34.2%) were married. Concerning their religion, 244 (64.7%) were Muslim, Protestant 54 (14.3%), and 79(21%) were Orthodox. Regarding their educational status, more than one-fourth of the study participants 167 (44.3%) were attending primary school followed by 74 (19.6%) unable to read and write. Concerning their residency and working status, nearly two-thirds of the study participants 236 (62.6%) were urban dwellers and 128 (34%) were students followed by 85 (22.54%), housewife 53 (14.1%), civil servants, and 49 (13%) were traders (Table 1).

**Table 1:** Socio- demographic characteristics of road traffic accidents admitted patients at Dessie Town governmental hospitals Northeast Ethiopia, 2022 (*n* = 377)

Variable	Category	Frequency(*n*)	Percent (%)
Sex	Male	237	62.9
	Female	140	37.1
Age group	*≤* 20	121	32.1
	21–40	182	48.3
	> 40	74	19.6
Marital status	Married	129	34.2
	Single	248	65.8
Religion	Muslim	244	64.7
	Orthodox	79	21
	Protestant	54	14.3
Education	Unable to read and write	74	19.6
	Primary school	167	44.3
	Secondary school	69	18.3
	College and above	67	17.8
Residence	Urban	236	62.6
	Rural	141	37.4
Occupation	Student	128	34
	Daily labor	27	7.2
	Farmer	26	6.9
	Trader	49	13.0
	Civil servant	53	14.1
	Deriver	12	3.2
	House wife	17	4.5
	Construction worker	33	8.8
	Unemployment	32	8.5

### Nature of the injury, diagnosis, their location, and Hospital name

The finding of this study showed that, all most three fourth of victims 281 (74.5%) were attended at Dessie Comprehensive Specialized Hospital followed by Boru Media General Hospital 96 (25.5%). Concerning the place where the victims came to hospitals 159 (42.2%) of the victim came from health centers followed by 131 (34.7%) from primary Hospitals, and 87 (23.1%) from the scene. Regarding of region of injury, musculoskeletal (lower extremities) 80 (21.2%) were the most affected region of the body followed by the Chest 80 (18.6%), head and neck 67 (17.8%), abdomen 51 (13.5%), upper extremities 38 (10.1%), bone fracture 33 (8.75%), and more than one parts of the body 28 (7.4) were accounting of the cases. Concerning the diagnosis of cases, internal organ injuries 108 (28.6%) was the most diagnosed injury followed by fracture and dislocation 96 (25.5%), head injury 51 (13.5%), soft tissue injury (Bruise, abrasion, laceration) 50 (13.3%), multiple organ injuries 49 (13%), and 23 (6.1%) were spinal cord injury (Table 2).

**Table 2:** Nature of the injury, diagnosis, their location, and Hospital Name related to road traffic accidents admitted patients at Dessie Town governmental health facilities Northeast Ethiopia 2022 (*n* = 377)

Variable	Category	Frequency(*n*)	Percent (%)
Hospital Name of pt. admitted	Dessie Comprehensive specialized hospital	281	74.5
	Boru Media general hospital	96	25.5
The place where the victim come to Hospital	From health center	159	42.2
	From primary hospital	131	34.7
	From the scene	87	23.1
Body region injured	Musculoskeletal (Lower extremities)	80	21.2
	Chest	70	18.6
	Neck and Head	67	17.8
	Abdomen	51	13.5
	Upper extremities	38	10.1
	Bone fracture	32	8.5
	Multiple body part	28	7.4
Main Diagnosis	Internal organ injuries	108	28.6
	Fracture and dislocations	96	25.5
	Head injury	51	13.5
	Soft tissue injury	50	13.3
	Multiple organ injuries	49	13.0
	Spinal cord injury	23	6.1

### Vehicle type that was involved in the accident and patient condition related to road traffic accidents

This study showed that 111 (29.4%) injuries were caused by Bajaj followed by, 91 (24.1%) by Motor cycle, 61 (16.2%) by Taxi, 44 (11.7%) by a heavy trucks, 28(7.4%) by Pickup, and the remained caused by Bus, Minibus, and other vehicles. Concerning the admission ward, nearly half of the road traffic victims 180 (47.7%) were admitted to the surgical ward followed by 106 (28.1%) in the ICU ward, 71 (18.8%) in the paediatric ward, and 20 (5.3%) were admitted in the medical ward. Concerning Hospital treatment more than half percent of the victims 217 (57.6%) were gate pre Hospital treatment and stable Vital sign were saw among 222 (58.9%) road traffic victims during admission more than 158 (41.9%) of the road traffic victim were severely injured followed by 139 (36.9%) Moderately injured, and 80 (21 0.2%) were had minor injury related road traffic accidents. Concerning the mental status of the victim 160 (42.4%) had a GCS range of 3–8 followed by 141 (37.4%), a GCS range of 9–12, and 76 (20.2%) with a GCS range of 13–15. Regarding comorbidity disease 120 (31.8%) victims had comorbidity diseases. Regarding hospital length of stay, 168 (44.6%) stayed at the hospital for less than one-month duration followed by 104 (27.6%) were stayed for one to two months duration, 70 (18.6%) stayed for three to months duration, and 35 (9.3%) stays for more than three months duration in the hospital this including the day spent for follow up after they were discharged (Table: 3).

**Table 3:** Ward and patient condition related to road traffic accidents admitted patients at Dessie Town governmental health facilities, Northeast, Ethiopia 2022 (*n* = 377)

Variables	Category	Frequency(*n*)	Percent (%)
Type vehicle that involved the accident	Bajaj	111	29.4
	Motor cycle	91	24.1
	Taxi	61	16.2
	Heavy truck	44	11.7
	Isuzu	28	7.4
	Minubace	15	4
	Bus	15	4
	Other	12	3.2
Type of ward the victim admitted	ICU	106	28.1
	Surgical	180	47.7
	Pediatric ward	71	18.8
	Medical ward	20	5.3
Pre Hospital Treatment	Yes	217	57.6
	No	160	42.4
Vital sign at admission	Stable	222	58.9
	Unstable	155	41.1
Degree of injury	Severe	158	41.9
	Moderate	139	36.9
	Minor	80	21.2
Glasgow coma scale	3–8	160	42.4
	9–12	141	37.4
	13–15	76	20.2
Comorbidity disease	Yes	120	31.8
	No	257	68.2
Length of Hospital stay	< 1 month	168	44.6
	1month − 2 month	104	27.6
	>=3 month	105	27.9
Treatment out come	Improved	136	36.1
	Discharged with disable	97	25.7
	Died after intervention (Air way opening technique, putting cervical collar, blood transfusion, Etc.)	62	16.4
	Immediately died (time less than one hours)	61	16.2
	Referred to higher level	16	4.2
	The result was not known	5	1.3

### Magnitudes related to road traffic accidents

**Fig. 3:**
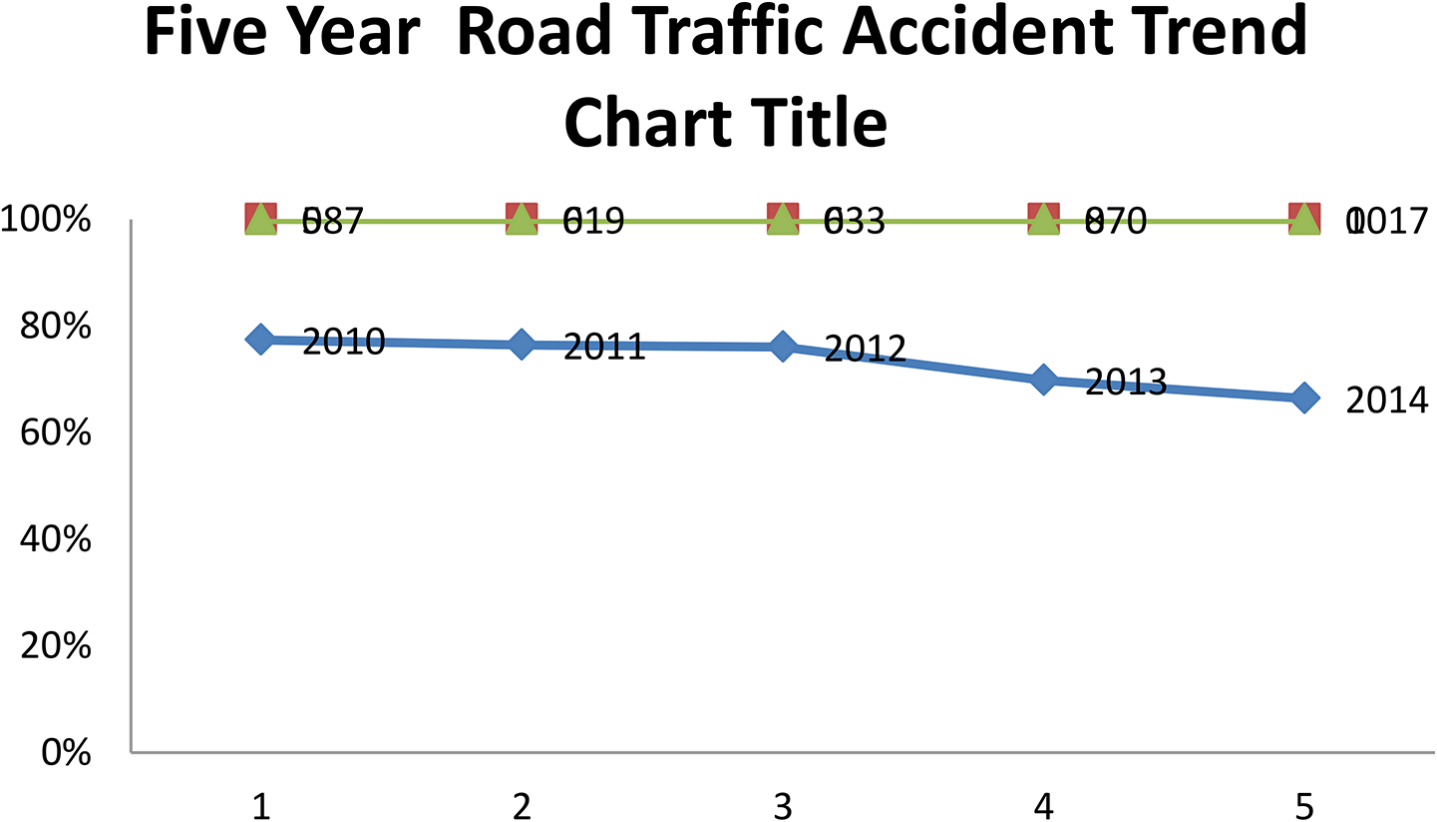
Road traffic accident trends in the 5 years at Dessie Town governmental hospitals from June 7/2022 to May 2017 Northeast,2022 (*n* = 377)

In this study, 377 road victims participated at Dessie Town governmental Hospitals with the magnitude of road traffic accidents found to be 59% throughout 5 years at DessieTown governmental Hospitals showed that the road traffic accidents dramatically increased from the year 2010 until the year 2014 according to Ethiopian calendar (figure 3).

### Factors associated with death

To assess the association of different independent variables with treatment outcome, bivariable multinomial logistic regression analysis was conducted for a crude association, and all variables with a (P-Value *≤* 0.2) were candidates for multivariable multinomial logistic regression. unstable vital sign at admission (AOR = 6.4,95%CI; 2.5–16.6), unable to gate prehospital treatment (AOR = 9.3,95% CI; 4–20), and severee injury (AOR = 9, 95% CI;7-15.4), and had Glasgow coma scale 3–5 (AOR = 5.2,95%CI; 1.4–20) were found predictors for death among road traffic admission victims.

### Factors associated with disabilities related to road traffic accident victims

The findings of this study shows that unstable vital signs at admission (AOR = 3.79, 95%CI;2.1–6.8,), Do not getting Hospital treatment (AOR = 2.8, 95% CI; 1.4–5.7), Hospital stay for one to two months duration (AOR = 6,95% CI;2.3–15), greater than two months duration (AOR = 6.5,95%CI;2.5–17) were found predictors for disability among road traffic accident admission Victims (Table 4).

**Table 4:** Bivariable and multivariate logistic regression results among road traffic accidents admitted patients at Dessie Town governmental hospital Northeast, Ethiopia 2022 (*n* = 377)

	Treatment outcome, improved as base outcome								
	Improved	Death	Disabled	Death		*P*-Value	Disabled		*p*-value
				COR (95%,CI)	AOR (95%,CI)		COR (95%,CI)	AOR (95%,CI)	
Sex									
Female	50 (35.7%)	54 (38.6%)	36 (25.7%)	1			1		
Male	101 (42.6%)	61 (25.7%)	75 (31.7%)	0.56 (0.34.92)	0.5(0.22-1.18%)	0.11	1.03(0.61- 1.73)	1(0.48-2.06)	1.00
Age									
<=20years	58 (38.4%)	39 (33.9%)	24 (21.6%)	1.06 (0.50-2.21%)	2.5(0.37- 17.84)	0.33	0.37(0.18.75)	0.59(1.44- 2.24)	0.59
21–40 years	66 (43.7%)	59 (51.3%)	57 (51.4%)	1.42(0.7-2.9)	1(0.29 − 3.5%)	0.98	0.77(0.41- 1.45)	0.62(0.25-1.55)	0.62
>40 years	27(17.9%)	17 (14.8%)	30 (27%)	1			1		
Marital status									
Single	85(56.3%)	73 (63.5%)	90 (81.1%)	1.35 (0.8-2.2)	3.35(0.76-14.59)	0.1	3.3(1.85.5.9)	2.5(0.85 − 7.3)	0.09
Married	66(43.7%	42 (36.5%)	21 (18.9%)	1			1		
Education status									
Unable to read and write	35(23.2%)	18(15.7%)	21(18.9%)	0.44(0.21-0.9)	0.54(0.16 − 1.8)	0.32	0.45(0.23-0.89)	0.21(0.07–0.58)	0.27
Primary school	77 (51%)	52(45.2%)	38(34.2%)	0.45(0.22-0.89)	0.9(0.32 − 2.6)	0.87	0.37(0.21-0.65)	0.7(0.32-1.67)	0.41
Secondary school and above	39(25.8%)	45(39.1%)	52(46.8%)	1			1		
Residence									
Urban	85(56.3%)	77(67%)	74(66.7%)	1.57(0.95 − 2.60)	0.75(0.34 − 1.6)	0.46	1.55(0.93 − 2.5)	1.48(0.77 − 2.8)	0.23
Rural	66(43.7%)	38(33%)	37(33.3%)	1			1		
Occupation									
Daily labor	52(34.4%)	36(31.3%)	33(29.7%)	0.79(0.46 − 1.3)	0.95(0.34—2.6)	0.95	1(0.57 − 1.7)		
Civil servant	18(11.9%)	8(7%)	27(24.3%)	0.5(0.2-1.2)	0.66(0.14-2.99)	0.59	2.3(1.1–4.75)		
Unemployed	81(53.6%)	71(61.7%)	51(45.1%)	1			1		
Name of Hospitals									
Boru media GH	39(25.8%)	33(34.4%)	24(25%)	0.8 (0.5-1.5)	1.1(0.42 − 2.9)	0.81	1.2(0.7-2.2)	0.89 (0.43 − 1.8)	0.76
Dessie CSH	112(74.2%)	82(71.3%)	87(78.4%)	1			1		
Vital sign at admission									
Stable	128(84.8%)	28(24.3%)	66(59.5%)	1			1		
unstable	23(15.2%)	87(75.7%)	45 (40.5%	17.2(9.3 (31.9)	3.5(1.9–7.8)	6.4(2-16.6)	0.000	3.79(2.1–6.8)	0.002
Pre Hospital treatment									
Yes	122(56.2%)	23(20%)	72(64.9%)	1			1		
No	29(19.2%)	92(80%)	39(35.1%)	16 0.8(9.1–31)	9.3(4–20)	0.000	2.2(1.29–3.99)	2.8(1.4–5.7)	0.004
GCS									
3–8	36(23.8%)	78(67.8%)	44(39.6%)	13 (5.9–29.7)	5.2(1.4–20)	0.001	5(2.4–10.9%)	1.8(0.6-5.4)	0.26
9–12	60(39.7%)	28(24.3%)	54(48.6%)	2.8 (1.2–6.5)	4.7(0.9–17)	0.09	3(1.8–7.7)	1.6(0.6-4.4)	0.32
13–15	55(36.9%)	9(7.8%)	13(11.7%)	1			1		
Management type									
Medical Rx	91(60.3%)	36(31.3%)	63 (56.8%)	0.3(0.18-0.50)	0.3(0.13-0.75)	0.09	0.86(0.52 − 1.4)	0.5(0.28 − 1.1)	0.101
Surgical Rx	60(39.7. 6%)	79(68.7%)	48(43.2%)	1			1		
Length of hospital stay									
Less than one month	57(37.7%)	100(87%)	11(9.9%)	1			1		
One –two months	45(29.8%)	7(6.1%)	52(46.8%)	0.09(0.04-0.21)	0.15(0.5-0.45)	0.001	5(2.3–10.8)	6(2.3–15	0.000
Greater than 2 months	49(32.5%)	8(7%)	48(43.2%)	0.09(0.03-0.21)	0.12(0.04-0.38)	0.000	5(2.8–12.8)	6.5(2.5–17)	0.000
Body region injured									
Head, neck injuries	43(28.5%)	48(41.7%)	7(6.3%)	0.09(0.41-0.21)	0.85(0.27 − 2.6)	0.78	5(2.3–10.8)	0.18(0.06-0.5)	0.002
Central body injuries	51(33.8%)	37(32.2%)	49(44.1%)	0.089(0.038-0.21)	0.72(0.26 − 2	0.53	5.9(2.8–12.7)	1.6(0.82 − 3.4)	0.151
Extremities injuries included fracture	57(37.7%)	30(26.1%)	55(49.5%)	1			1		
Degree of injury									
Sever	50(33.6%)	79 (53%)	20(13.4%)	8.69(3.4–22)	9(7-15.4	0.001	0.28(0.142-0.56	0.3(0.6-0.84	0.12
moderate	68(47.9%)	30(21.1%)	44 (31%)	2.42 (0.92 − 6.4)	1.5(0.95 − 3.4	0.56	0.45(0.25-0.81)	0.4(0.58 − 6)	0.34
Minor	33(38.4%)	6(7%)	47(54.7%)	1			1		

COR = P value ≤ 0.25 variables were exported to multivariate multi nominal logistic regression AOR = P Value ≤ 0.05 show significant association

## Discussion

This study revealed that the prevalence of road traffic accidents was found to be 59%.

This result is in line with the study carried out in Yirgalem General Hospital, Southern Ethiopia with the prevalence reported as (51.4%) [16]. in Wolaita Zone, SNNPR, Ethiopia where the prevalence was 62.5% [17], in Adama Hospital Medical College, Central Ethiopia where the prevalence was reported as 54.7% [10].

On the other, the present study finding was higher than the studies done in the emergency departments of the University of Gondar Comprehensive Teaching and Referral Hospital (UOGCTRH) which was found as 33.6% with (95%CI: 28%, 39.1%) [14]. In the Emergency Department at Tikur Anbessa Specialized Referral Hospital, Addis Ababa, Ethiopia the prevalence was reported as (38.3%) [18].

The variation of the prevalence for the UOGCTRH study might be due to the study period which was only for 6 months duration and it was limited to the specific departments which was done in the emergency department only and the prevalence variation for Tikur Anbessa Specialized Referral Hospital was the first thing was the duration of the study done for only three months duration and also it was area specific which were done in the emergency department.

On the contrary, the prevalence of road traffic accidents was lower than in the study done in the Emergency Department of Tikur Anbessa Specialized Teaching Hospital, Addis Ababa, Ethiopia where the prevalence was reported as 74% [19]. The variation of the prevalence for this study might be due to the study area where conducted in Addis Ababa Ethiopia, the most transportation-covered area since the town is the capital city of Ethiopia. The study was conducted in Saudi Arabia (84.4%) [20], in Vellore district, southern India (73%) [21], and, in Diredawa, Eastern Ethiopia (80%) [22].

Regarding associated factors, road traffic accident victims who have unstable vital signs at admission are six times more likely to die as compared to clients who have stable vital signs (AOR = 6.4, 95%CI; 2.5–16.6). Associated between unstable vital signs and death among traumatic patients was reported in a study done at the Adult Emergency Department of Tikur Anbessa specialized hospital, Addis Ababa, Ethiopia which states that systolic blood pressure which is one of the vital signs was a statistically significant predictor of fatalities among the road traffic victims [7]. Moreover clients unable to gate pre Hospital treatment have nine times more likely to die as compared to client gate pre Hospital treatment (AOR = 9.3,95% CI; 4–20). This finding was supported by the study done by Bugando Medical Centre in Northwestern Tanzania [23]. Which states that prehospital care is a very important factor in determining the outcome after injury [24]. In the current study clients who have severe injury were nine times more likely to die as compared to clients who have minor and moderate injury (AOR = 9, 95% CI;7-15.4). The finding of the study was supported by a study done at a Tertiary Hospital in Kenya [6]. and at Bugando Medical Centre in Northwestern Tanzania as reported as a High mortality rate was recorded in patients with severe trauma at admission [22]. Finally, road traffic victims having a Glasgow coma scale of 3–8 were five times more likely to die as compared to road traffic victims having a Glasgow coma scale of 9–12 and 13–15 (AOR = 5.2,95% CI; 1.4–20). This result was supported by a study done in Jimma Ethiopia [8]. This states that patients with low GCS are highly liable for a bad outcome that could be due to major organ failure, especially severe head injury.

Regarding factors associated with disabilities related to road traffic accident victims.

The current study discovered that unstable vital signs at admission (AOR = 3.79, 95% CI; 2.1–6.8) was nearly 4 times more likely to develop a physical disability, this study results in line with the study done in Dar es Salaam, Tanzania [25]. The other concern related to road traffic victims who Don’t get pre-Hospital treatment were nearly three times more likely to develop functional disability as compared to road traffic victims who got pre-hospital treatment (AOR = 2.8, 95% CI; 1.4–5.7) this finding was supported by the study done in Tikur Anbessa specialized hospital, emergency department [26]. as stated as It is obvious that primary prevention is the best way to avoid or to reduce rates of death or disability from a life-threatening injury. Moreover, Hospital stay for one to two months duration (AOR = 6,95% CI; 2.3–15) and greater than two months duration (AOR = 6.5,95% CI; 2.5–17) were nearly seven times more likely to develop physical disability as compared to clients who stay in hospital Less than one-month duration among road traffic accident Victims this founding was supported by a study done in China as state that performance of daily activities were associated with prolonged hospital stay [27].

### Limitation

Since the current study was cross-sectional, this is weak to evaluate the cause–effect relationship also the current study depending to the client’s chart review some important information like the victim’s time spent before rich to the hospital and substance use was difficult to access.

## Conclusion

The findings of this study showed that the magnitude of road traffic accidents was found to be 59%. Unstable vital signs at admission, Don’t getting pre-Hospital treatment, Hospital stay for one to two months duration, and greater than two months duration were associated.

### Recommendation

The health care providers working in the respective ward and unit care service better give strong attention to road traffic victims those having unstable vital signs at admission, unable to get pre Hospital treatment, sever injury, and victims having a Glasgow coma scale of 3–8, and Hospital stay for more than one month duration. Better treatment outcome including disabilities free road traffic victims.

It is better incorporated in routine follow up on community awareness program on rood traffic safety. To assess Magnitude and outcome of Road traffic accident among Patients Admitted to Dessie town Governmental Hospitals, are better evaluated in prospective study design.

### Electronic supplementary material

Below is the link to the electronic supplementary material.

Supplementary Material 1

## Data Availability

The datasets used and/or analyzed during the current study are available from the Corresponding author upon reasonable request.

## Abbreviations

AOR: Adjusted Odds Ratio
BSC: Bachelor of Sciences
CI: Confidence Interval
GCS: Glasgow Coma Scale
RTA: Road Traffic Accident
RTI: Road Traffic Injuries
WHO: World Health Organization
