# Supplementary material for: Magnitude and outcome of road traffic accidents among patients admitted in dessie town governmental hospitals, Northeast Amhara, Ethiopia, 2022
Source: BMC Emerg Med. 2024 Jul 29;24:138. doi: 10.1186/s12873-024-01047-1 (PMC11288062; doi:10.1186/s12873-024-01047-1)
Supplement: Supplementary file 1 — Supplementary Material 1 [file 12873_2024_1047_MOESM1_ESM.docx]

**ANNEXES**

**Annex 1:** Information extraction format

**Part I:** Socio-demographic characteristics of the study participants on the magnitude of road traffic accidents, treatment outcome, and associated factors at Dessie Town governmental Hospitals, Northeast Ethiopia, 2022.

| CODE -------------------------DATE----------------- | | |
| --- | --- | --- |
| 101 | Age | In ----------------------years |
| 102 | Sex | 1.Male  2. Female |
| 103 | Marital status | 1.Unmarried  2. Married  3.Divorced  4.Widowed  5.Cohabiting  6 .Not applicable |
| 104 | Educational status of the Victim | 1.Unable read and write  2.Primery school  3.Secondry school  4.Deploma and above |
| 105 | Resident | 1. Urban  2. Rural |
| 106 | Religion | 1.Orthodox  2.Muslim  3.Protestant  4.Others specify ------------------------ |

**Part II**: Effects of injury on the body part related questionnaires

| No. | Questions | Category |
| --- | --- | --- |
| 201 | Time of the accident | In time -----Day------month--------year------- |
| 202 | Time of hospital admission | In time ------day-----month----and year |
| 203 | The place where the victim come to the Hospital | 1.The scene  2. Hospital  3. Health center |
| 204 | Type of ward admitted | 1.Medical ward  2.Surgical ward  3.Pediatric ward  4.ICU ward  5.Other ward specified ________ |
| 205 | Major body region involved | 1.Head and neck  2.Chest  3.Abdomen  4.Pelvic  5.Upper extremities  6.lower extremities  7.Bone fracture  8.Other body region specify |
| 206 | Types of injury (main diagnosis) | 1.Soft tissue injury (Bruise ,abrasion , laceration )  2.Fracture and dislocations  3.Internal organ injuries  4.Head injury  5.Spinal cord injury |
| 207 | Patients vital sign at admission | 1.Vital sign stable  2.Vital sign unstable |
| 208 | Degree of injury | 1.Minor |
|  |  | 2.Moderate |
|  |  | 3.Severer |
| 209 | GCS | 1.3-8 |
|  |  | 2.9-12 |
|  |  |  |
|  |  | 4.Not determined |
| 210 | Type of management | 1.Medical  2. Surgical  3.Medical and Surgical  4.Conservative treatment |
| 211 | Treatment out come after admission | 1.Immediately died  2.Died after intervention  3. Improved  4.Discharged with Disabled  5.Referred to higher level  6.The result was not known |
| 212 | Time to discharge | In time ------day------------month ---------and -------years |
| 213 | Hospital stay (LoS) | In day----------month-------and years-------- |

**Annex 2. Information sheets for Hospital administration**.

I want to collect data for the study that conducted in Dessie Town governmental Hospitals. I am a lecture at Wollo University College of medicine and health Sciences. I kindly request you to lend me your attention to explain you about the study and your Hospital being selected as the study site.

**The study title**: Magnitude of road traffic accidents and treatment outcome and associated factors in Dessie Town governmental Hospitals

**Purpose of the study:** The findings of this study can be of a paramount importance for the Hospital to plan intervention programs for Road traffic accident victims and to increase basic surgical, medical, and orthopedics service and to decrease injury related mortality and disabilities rate in general.

I was collected data from surgical, medical, and orthopedics, operation logo book, patient cards and charts using data extraction format to provide me with pertinent data that is helpful for the study. The data collection was taking about 60 days, so we kindly request you to have cooperation with the Hospital staff during data collection period.

**Risk and benefits**: There is no risk rather you only taking few hours for the staff for collecting patients’ charts.

**Confidentialit**y**:** The information collected in the patient chart was confidential. There was no information that was identifying patients in particular, to do so; each patient information was having a code. The findings of the study was general for the study population and was not reflect anything particular of individual person or patient.The data extraction format was coded to exclude showing names.

**Rights**: This study will be done if you are voluntary on the behalf of the hospital. You have the right to allow or not to allow this study in your Hospital. You have the right to stop this study if you observe any misconduct during data collection

Contact address: Mobile number **+251914074466**

**Email: fahmseid10@gmail.com**
